# Friendship and Loneliness Among People Living With Dementia: Social Practices and Identity

**DOI:** 10.1093/geroni/igab046.1385

**Published:** 2021-12-17

**Authors:** Pamela Saunders, Daniel R Y Gan, John Swinton

## Abstract

More people living with dementia (PLWD) are aging in place in the community. The number of PLWD aging in community is estimated to comprise 61-81% of the total number of PLWD in North America. Since most PLWD do not drive (Foley et al., 2000), many may (or may not) spend much of their time closer to home, barring occasional visits out of town. Yet, one’s everyday environment may not always provide “ways of being in the world that are more accepting and embracing” (Hillman & Latimer, 2017) and kind, to the varied socio-cognitive struggles of PLWD. Meaningful relationships are required to support continued social participation and citizenship (Bartlett & O’Connor, 2007; Swinton, 2020). In addition to dementia diagnosis, these everyday experiences in community may significantly alter PLWD’s self-perception and confidence. PLWD may feel more or less comfortable forging relationships depending on their past experiences. In other words, the identity of PLWDs are often challenged and (re)constructed (Saunders et al., 2011). Amid persistent power imbalances, malignant social practices may reshape one’s identity such that social isolation, whether self-imposed and/or due to restrictions from others, appear the best way to tide over overwhelming loneliness. This symposium explores how community and friendships may intercept the formation of such lonely self-identity among PLWD. We use advanced qualitative methods to elucidate the varied experiences and challenges of PLWD in community. Findings from three perspectives, namely sociolinguistics, sociology, and social work, will be discussed identify new social practices to undo stigma and support PLWD in community.

